# Electronic Processes at the Carbon-Covered (100) Collector Tungsten Surface

**DOI:** 10.3390/mi13060888

**Published:** 2022-05-31

**Authors:** Harilaos J. Gotsis, Naoum C. Bacalis, John P. Xanthakis

**Affiliations:** 1 Electrical and Computer Engineering Department, National Technical University of Athens, 15700 Athens, Greece; hgotsis67@yahoo.com; 2 National Hellenic Research Foundation, Vasileos Constantinou Str., 11635 Athens, Greece; nbacalis@eie.gr

**Keywords:** carbon-covered tungsten surface, workfunction, VASP calculation, scanning microscopy, tunnel diode

## Abstract

We have performed density functional VASP calculations of a pure and of a carbon-covered (100) tungsten surface under the presence of an electric field **E** directed away from the surface. Our aim is to answer the question of an increased penetrability of electrons at the collector side of a nanometric tunnel diode when covered by carbon atoms, a purely quantum mechanical effect related to the value of the workfunction Φ. To obtain Φ at a non-zero electric field we have extrapolated back to the electrical surface the straight line representing the linear increase in the potential energy with distance outside the metal-vacuum interface. We have found that under the presence of **E** the workfunction Φ = E_vac_ − E_F_ of the (100) pure tungsten surface has a minor dependence on **E**. However, the carbon-covered tungsten (100) surface workfunction Φ(C − W) has a stronger **E** dependence. Φ(C − W) decreases continuously with the electric field. This decrease is ΔΦ = 0.08 eV when **E** = 1 V/nm. This ΔΦ is explained by our calculated changes with electric field of the electronic density of both pure and carbon-covered tungsten. The observed phenomena may be relevant to other surfaces of carbon-covered tungsten and may explain the reported collector dependence of current in Scanning Field Emission Microscopy.

## 1. Introduction

Several experiments at ETH [1] under the title Scanning Field Emission Microscopy have demonstrated that tungsten (110) surfaces covered by monolayers of several adatoms in various complicated structures (such as 12 × 15) acting as collectors under an external electric field show increased received current compared to the pure metallic tungsten substrate. The experiments referred to field emission in a nanometric tunnel diode operating in a point facing plane configuration in which the current was shown to increase by an epitaxial carbon or other adatom cover of the tungsten collector. A decrease in the collector workfunction was hence necessary to explain the increase in absorbed current. Whereas the decrease in the workfunction gives a macroscopic explanation of the phenomenon, the exact quantum mechanical processes remained unknown. Given that the increased current of electrons was observed for several adatom types, a question arises naturally: could a simpler such structure show such a similar feature and would then the underlying changes in the fundamental quantum mechanical property of charge density become more apparent and be easier to be understood? Additionally, could the effects of the presence of the electric field be easier to interpret? Thus, we decided to carry out quantum mechanical computations of the simpler W(001) structure, pure as well as covered by a carbon monolayer under an external electric field pushing the electrons into the W(001) surface, a simulation of the collector in the above experiments. We used the standard package VASP for that matter. It transpires that the workfunction change could indeed be attributed and explained by simple changes in the quantum mechanical electron density. The nature of the latter near the C-covered W(001) surface, as will be shown, is such that it facilitates by Coulomb attraction the incoming electrons in penetrating the surface, whereas near the pure W(001) surface it, comparatively, does not, rather tending to repel the incoming electrons.

A very brief review on the relative importance of the emitter and collector workfunctions is necessary here. The original Fowler-Nordheim equation [2] referred to a planar capacitor configuration with a triangular barrier, the height of the triangular barrier being the emitter workfunction. An implicit assumption was that the collector was far away from the emitter with plenty of available empty states for electrons to occupy. This assumption was also taken by Nordheim in his later WKB treatment of the image-reduced triangular barrier [3] and by the classic treatment of Murphy and Good [4] in 1956. It was Simmons in 1963 [5,6] who realized that if the collector is near the emitter then the former may play a role in the emission. In particular, if the two electrodes—emitter and collector—have different workfunctions, then if the two are connected by a wire of infinitesimal resistance a voltage difference ΔV develops between the two electrodes of magnitude eΔV = ΔΦ = the workfunction difference. This is due to the necessary transfer of charge to equalize the Fermi levels. This voltage produces an electric field which increases or decreases the applied one depending on whether the collector workfunction is lower or higher, respectively, than the emitter one. Hence, the collector Φ was recognized as a factor in the emission in any nanometric diode configuration. Note that this argument holds true irrespective of the shape of the barrier, which in the case of the ETH experiments was the image-rounded triangular-like one.

The description given above is correct but not complete. In all the theoretical works mentioned above the Sommerfeld model for a metal was used and the workfunction of an emitter was taken as a constant characteristic of the metal under study. The same holds true for the relatively recent generalizations of Forbes [7] to nonmetals, the work of Jensen [8,9] on the unification of field and thermionic emission and that of Kyritsakis and Xanthakis [10] for the generalization to nanometric emitters. Jensen [11] in 1999 was the first to examine the possibility that the workfunction Φ of the emitter is electric field **E**-dependent. Explicit numerical calculations by Jensen [11] showed that the field contribution may be several tenths of an eV.

The above effects are expected to be more pronounced when foreign atoms are present on the investigated surface and may constitute the basis for the interpretation of the experiments mentioned in the Introduction. However, there is an additional complication in these experiments that deserves an explanation: whereas the current-voltage I-V measurements required a ΔΦ = −0.5 eV to be explained, Gundlach oscillations measurements [12] gave a value of ΔΦ = −0.1 eV. Such a difference could be explained if the collector workfunction was indeed field-dependent with the values of the collector workfunction at the two experiments (i.e., I–V and Gundlach oscillations) being different. It is therefore obvious that the electronic processes at the collector side of a nanometric diode are not clear and deserve investigation. The case of Field Emission Lithography [13] is another case where the electronic structure at the collector may be important. We note that such studies are very rare so far, despite the fact that situations where the anode-cathode separation of a few nm do exist and are important. The only such study is the calculation by Zhu et al. [14] for heavier metals which does not, however, show the link between the calculated ΔΦ and the change in the charge density.

In this paper, we examine the electronic processes at a tungsten (100) collector surface when it is pure and when covered by carbon that leads to the corresponding field dependence of its workfunction. We also note that the effect of carbon on the (100) surface of tungsten as an emitter has recently been investigated [15] but the present work investigates phenomena at the collector side.

## 2. Method

**Figure 1:**
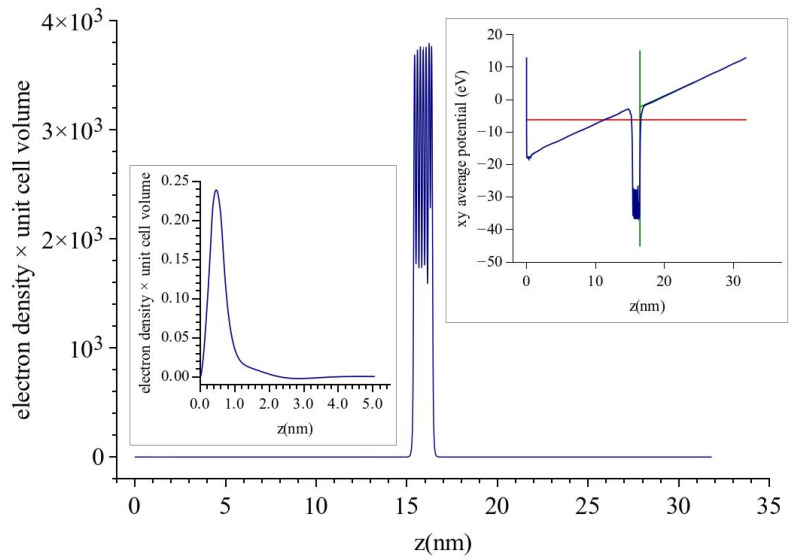
Laterally averaged electron density of seven layers of pure tungsten under an electric field of 1 V/nm. The electric field is applied so that it pushes in the electrons at the right hand side (RHS) surface. Near z = 0 a minute charge remains (see LHS inset) which does not affect the RHS surface. The RHS inset shows the form of the potential used by VASP. In all subsequent figures, the applied field is in the same direction.

We have not used analytical methods as in [11] but have resorted to numerical ab initio DFT calculations. We have used the program VASP for this task. According to the VASP prescription, a 2-dimensional periodic unit cell (along x, y) of the material under study along with a finite number of layers perpendicular to x, y (along z) is placed between the two plates of a capacitor which simulate the applied electric field. The system is allowed to relax according to the DFT equations which are solved under periodic boundary conditions [16]. However to preserve the periodic boundary conditions, the linear potential at its lowest point is abruptly increased to the high value at the other end of the unit cell producing a sawtooth-like potential. A picture of this is shown as an inset in Figure 1. In our study, pure and C-covered tungsten were represented by seven atomic layers. Only the front three layers, i.e., where **E** is pushing the electrons into the surface, were allowed to relax although no changes were recorded beyond the second layer. Note that due to the periodic boundary conditions, a triangular well is formed in front of the charge layer facing the back surface where electrons are pulled out of the metal. The energy cut-off for the expansion of the wavefunction was 317 eV and the condition for convergence of lattice geometry was that the change in the cohesive forces was less than 0.01 eV/A^0^. Purely electronic iterations were terminated when the difference in energy was less than 10^−4^ eV. Convergence of the DFT-VASP calculations are in general difficult as other workers have noted [17] and it is achieved by starting from zero electric field and then increasing it in very small steps of 0.1 V/nm. We have reached a maximum **E** of 1 V/nm. Unfortunately, we were not able to go to higher fields, but it transpired that this was enough to draw our conclusions. We note that when convergence has been achieved, a minute electron density corresponding to 1/100 of an electron remains in the triangular well in front of the back charge layer and a corresponding charge is missing from the back tungsten surface, see Figure 1 and insets there. This charge density is placed back on the back surface of tungsten and the effects of the transfer are taken into account by simple electrostatics. This refinement does not affect the front surface of the metal under study where the electric field is pushing the electrons into the surface and where our calculations are focused.

## 3. Results-Discussion

**Figure 2:**
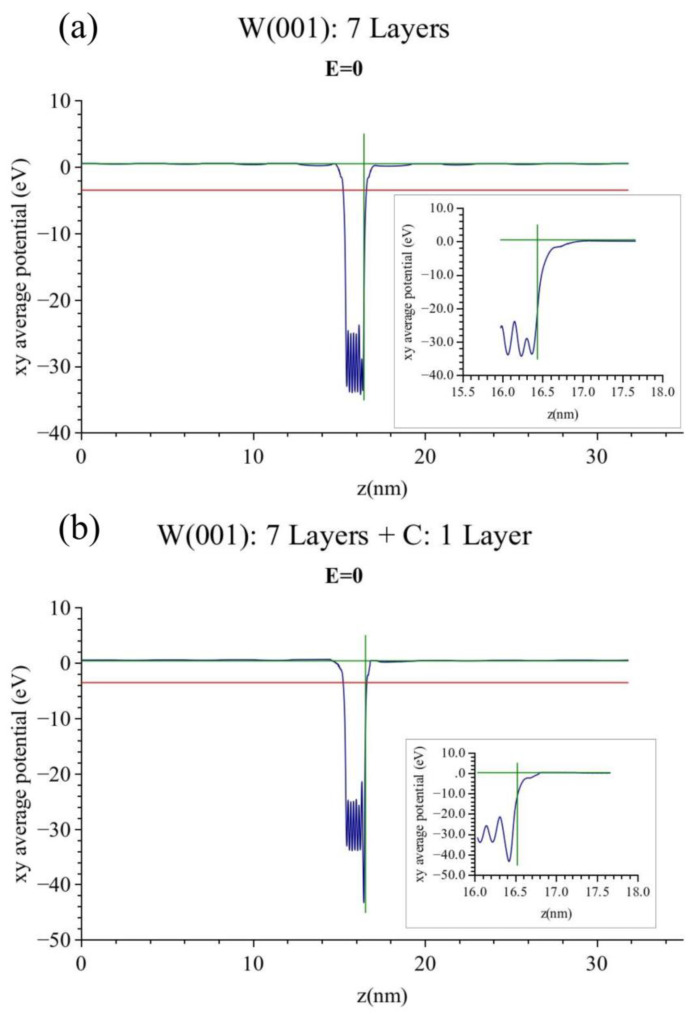
(**a**) Laterally averaged electron potential of pure tungsten with no applied electric field. The workfunction is 3.98 eV. The Fermi level is denoted by a red line as in all subsequent figures. (**b**) Laterally averaged electron potential of carbon-covered tungsten with no applied electric field. The workfunction is 3.93 eV.

The calculated band diagram of the clean (100) tungsten surface at ε = 0 is shown in Figure 2a. The clean (pure) tungsten workfunction is calculated to be (E_vac_ − E_F)_ = 3.98 eV. This value is smaller than the experimental value of 4.3–4.4 eV but this is mostly due to the Perdue exchange and correlation (XC) potential used in the present version of VASP. For the variation of the calculated tungsten workfunctions of most tungsten surfaces with respect to the XC functional used see [18]. The corresponding band diagram of the carbon covered tungsten is shown in Figure 2b From that diagram, the result comes out as (E_vac_ − E_F_) = 3.93 eV, i.e., 0.05 eV lower. The previous comments of Figure 2a, referring to the XC functional, also apply here.

**Figure 3:**
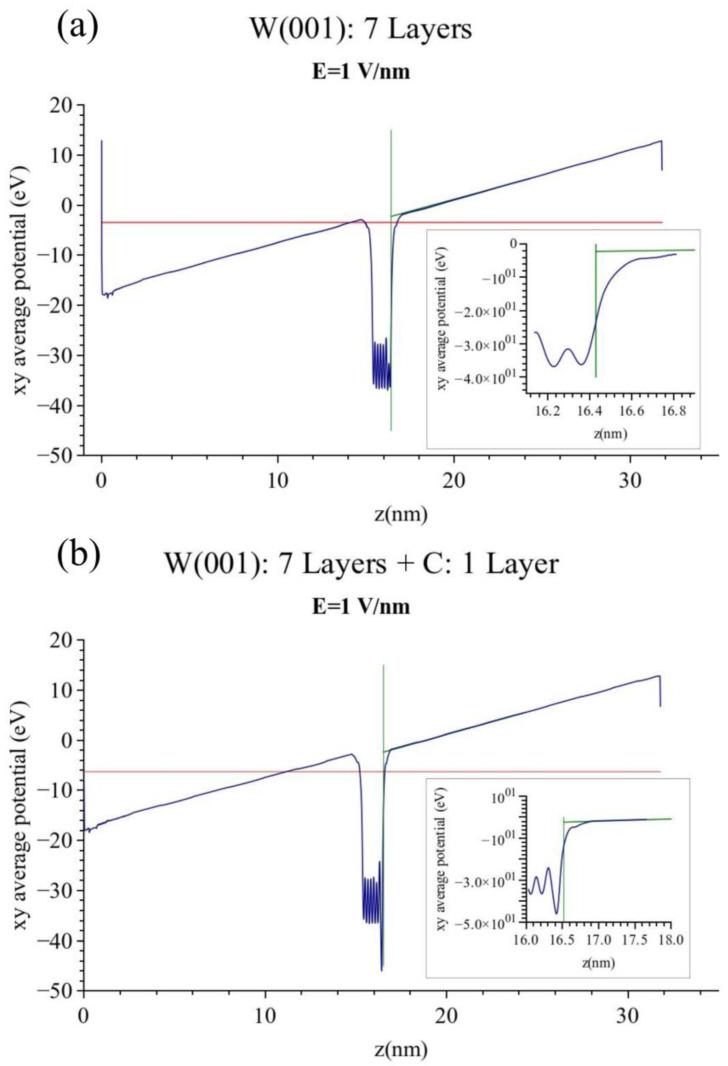
(**a**) Laterally averaged electron potential of pure tungsten with an applied electric field of 1 V/nm. The workfunction is 3.98 eV the same as with no field. (**b**) Laterally averaged electron potential of carbon-covered tungsten with an applied electric field of 1 V/nm. The workfunction is 0.08 eV lower that at zero field.

We now proceed to the band structure calculations of the clean and carbon-covered (100) tungsten surfaces when an electric field is pushing the electrons into the solid. A problem arises in these cases with the definition of the work function. The quantity (Evac-EF) cannot be used immediately since there is no constant vacuum energy outside the metal surface under investigation, see Figure 3a which gives our calculated band structure of the clean (100) tungsten surface at **E** = 1 V/nm. Instead the energy at every point outside the W (100) surface increases linearly in accordance with the applied electric field. The answer to this problem can be found in the work of Lang and Kohn [19] and Forbes [20] on the definition of the electrical surface. We remind the reader that this is the surface from which the electric field begins to act and it was shown to be the centroid of the induced surface charge. This is usually taken to be half the interatomic distance away from the surface of the outer nuclei. We therefore extrapolate the straight line representing the increase in potential energy outside the investigated surface back towards this surface and define E_vac_ as the value of the VASP energy at the electrical surface, see Figure 3a,b.

Using the above definitions we observe from Figure 3a no appreciable change of the workfunction Φ(W) of clean tungsten at **E** = 1 V/nm. However, when we examine the corresponding figure of the carbon-covered tungsten surface, Figure 3b, we observe a decreased workfunction Φ(C − W) = 3.9 eV. This gives a change ΔΦ = Φ(W) − Φ(C − W) = 0.08 eV at **E** = 1 V/nm. We have verified that for values of the electric field up to the value of ε = 1 V/nm this change was proportional to the field. We expect therefore that at the more usual values of **E** = 4–5 V/nm at which field emission is performed the expected ΔΦ would be around 0.4 eV. This would be a substantial change of the workfunction of the carbon-covered tungsten collector surface by the field and it would affect exponentially any narrow gap diode. Bearing in mind the underestimates that the Perdue potential is producing of the workfunctions, ΔΦ might actually be higher.

**Figure 4:**
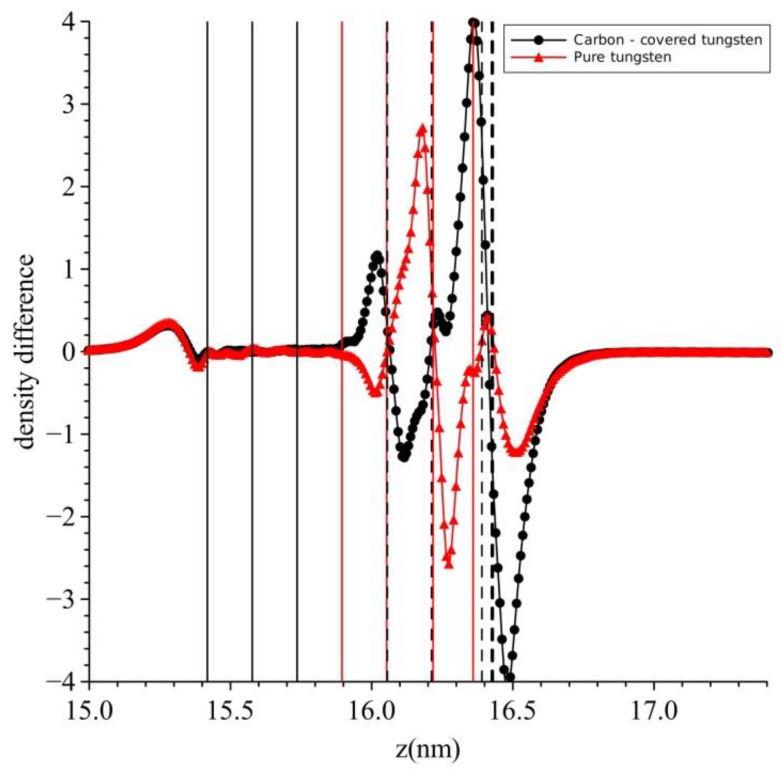
Difference in electron density between the cases of zero field and an electric field of 1 V/nm. Red line (triangles) is for pure carbon and black line (circles) for carbon-covered tungsten. See text for details.

The electronic processes responsible for the above changes are described in the following Figure 4 of the difference in electronic density Δρ(z) = ρ(z, **E** = 1 V/nm) − ρ(z, **E** = 0). The positions of the atoms are also shown in this figure. The red thin lines denote the atoms of pure tungsten. The rightmost dashed thicker black line denotes the carbon atoms of carbon-covered tungsten while the thinner black dashed lines denote the positions of the relaxed tungsten atoms of carbon-covered tungsten. In the case of pure tungsten, there is an accumulation of charge density (red line) just outside the plane of tungsten nuclei and also a depletion layer immediately further out from the accumulation one. Obviously the incoming electrons have “squeezed” the host electronic density into the metal, note that this diagram gives the change in electronic density. A similar “squeezing” occurs in the case of the carbon-covered tungsten (black line), only in this case the “squeezing” is more intense and its accumulation layer has moved into the metal, i.e., to the left of the surface nuclei. Therefore, the incoming electrons will find it easier to enter the metal since they will be penetrating a less charged region compared to pure tungsten. Hence, a reduction in the workfunction should be expected as it does. In a more traditional way, one can view this process as a reduction in surface dipole moment.

The above process could be expected to hold for all carbon-covered tungsten surfaces since they are the result of purely coulombic interactions. We tentatively argue that they may also be responsible for the observed decrease in the workfunction of the carbon-covered tungsten collector (110) surface in [1].

## 4. Conclusions

We have shown numerically that the workfuction of carbon-covered tungsten acting as a collector is electric field-dependent whereas that of pure tungsten is not to any significant value. This effect was shown to be the result of the reorganization of the electronic density of pure tungsten due to the presence of both the carbon adatoms and the electric field. The observed phenomena at the collector side might play a role in experiments where emission is observed to depend on the collector atomic structure.
